# The impact of mandatory physical fitness testing on students' intrinsic motivation and sustained participation in physical activity: a self-determination theory perspective

**DOI:** 10.3389/fpsyg.2026.1821930

**Published:** 2026-06-10

**Authors:** Ying Chen, Jiahang Jiang, Wenwu Liu

**Affiliations:** School of Physical Education and Health, East China Normal University, Shanghai, China

**Keywords:** physical education entrance examination, self-determination theory, autonomy, competence, relatedness

## Abstract

**Introduction:**

With physical education included in China's high school entrance examination, the high-stakes Physical Education Entrance Examination (PEEE) has altered school physical education arrangement. Previous studies mainly focus on physical fitness outcomes but lack in-depth exploration of motivational mechanisms from Self-Determination Theory (SDT). This research aims to examine how PEEE influences students' sport motivation and sustained physical activity participation by affecting their three basic psychological needs of autonomy, competence and relatedness.

**Methods:**

A qualitative approach was employed. Twenty-four junior high students and eight PE teachers from four geographically diverse middle schools were selected via purposive sampling. Data were collected through individual semi-structured interviews, focus group talks and classroom observations, and thematic analysis based on Braun and Clarke's framework was conducted with NVivo 12 software.

**Results:**

The PEEE increases institutional and parental attention to PE and improves short-term physical fitness and self-confidence for part of students. However, rigid exam-oriented courses limit students' autonomous choices; individual physical gaps result in polarized feelings of competence; score competition erodes supportive interpersonal relatedness. Most students form extrinsic exercise motivation instead of internalized interest, and teachers face persistent conflicts between educational ideals and exam assessment pressure.

**Discussion:**

Although the PEEE partially achieves fitness promotion goals, it distorts three core psychological needs proposed by SDT and blocks the internalization of sport motivation. To foster lifelong exercise behavior, future PE reform should replace unified high-stakes testing with diversified process evaluation and adopt autonomy-supportive teaching strategies.

## 1 Introduction

In recent years, China has promoted the physical health of adolescents by incorporating physical education into the High School Entrance Examination (hereafter “PE Entrance Exam, PEEE”) (Ministry of Education of the People's Republic of China, 2020). This policy requires students to participate in standardized physical fitness tests, which typically account for a certain proportion of the total exam score (Bai et al., 2022). As a high-stakes assessment, the PEEE aims to motivate students to improve their physical fitness and cultivate exercise habits. However, embedded within China's exam-oriented education culture—characterized by high external control and extrinsic incentives such as test scores—it raises questions about its impact on students' motivation. In this context, the mandatory nature of the PEEE may simultaneously promote physical activity participation while undermining students' intrinsic motivation and long-term interest in exercise.

Self-Determination Theory (SDT) provides a theoretical foundation for analyzing this phenomenon, emphasizing the importance of autonomy, competence, and relatedness in sustaining motivation (Deci and Ryan, 2008; Ryan and Deci, 2017). Although SDT has been widely applied in physical education, research in high-stakes and compulsory assessment environments remains limited, particularly within China's unique exam-oriented culture. In contrast, countries such as the UK and Australia emphasize formative assessment and student choice in physical education, highlighting the distinctiveness of China's PEEE. Existing research largely focuses on the exam's effects on physical health outcomes, while overlooking its deeper influence on students' intrinsic motivation and long-term participation in physical activity.

This study adopts a qualitative approach to explore how the PEEE influences students' intrinsic motivation and long-term willingness to participate in physical activity, from both student and teacher perspectives. Situated within China's highly controlling educational environment, this research aims to fill the gap in SDT applications in the context of mandatory physical assessments, provide insights for policy optimization, and contribute a cross-cultural perspective to global research in physical education.

## 2 Literature Review

### 2.1 Self-determination theory and its application in physical education

Self-Determination Theory (SDT), proposed by Deci and Ryan, posits that human motivation originates from the satisfaction of three basic psychological needs: autonomy, competence, and relatedness (Deci and Ryan, 1985, 2000). Autonomy refers to a sense of volition and choice; competence involves feeling effective in one's interactions; and relatedness concerns forming meaningful social connections. A core tenet of SDT is the internalization process, through which individuals transform externally prescribed values or regulations into personally endorsed ones (Ryan and Deci, 2000). Along the motivation continuum, SDT categorizes regulation from amotivation, through various forms of extrinsic motivation (external, introjected, identified, and integrated regulation), to intrinsic motivation. While intrinsic motivation—driven by inherent enjoyment—is the gold standard for behavioral persistence, identified and integrated regulations are crucial for sustaining activities that are not inherently “fun” but are personally valued (Vansteenkiste et al., 2006, 2010).

In physical education (PE), SDT research consistently shows that autonomy-supportive environments—characterized by providing choices and meaningful rationales—enhance intrinsic motivation and long-term adherence (Hagger and Chatzisarantis, 2008). For instance, Ntoumanis (2001) and Hagger et al. (2005) found that allowing students to choose activities or adjust intensity increased engagement and extracurricular participation. Similarly, competence-supportive practices, such as providing optimal challenges and positive feedback, are essential for fostering confidence and satisfaction (Spray and Wang, 2001; Taylor et al., 2010). Furthermore, a sense of relatedness fostered through team-based support and warm teacher-student relationships is a strong predictor of positive classroom experiences (Cox et al., 2008; Sparks et al., 2016).

However, a critical yet under-explored area is how these mechanisms function within high-stakes, standardized assessment contexts. International research on “accountability-driven” education suggests that high-stakes testing often acts as a “controlling regulator” that undermines the internalization process. When the focus shifts predominantly to scores, the “washback effect” can trap students in external regulation, preventing the transition to more autonomous forms of motivation (Reeve, 2009). While some studies in East Asia have noted that assessments can lead students to view PE merely as an “exam task” (Sun et al., 2017), there is limited systematic analysis of how China's intensive Physical Education Entrance Examination (PEEE) affects the quality of motivation.

By examining this highly exam-oriented context, the current study aims to contribute to SDT by clarifying the “theoretical boundary” of needs satisfaction: specifically, exploring whether gains in competence (e.g., higher test scores) can lead to genuine internalization when autonomy is systematically constrained. This adds a necessary cross-cultural dimension to SDT, highlighting the potential for “functional distortion” of psychological needs in high-pressure educational systems.

### 2.2 The uniqueness of China's PE entrance exam

China's PEEE—characterized by high stakes and standardization—occupies a distinctive position in global physical education assessment systems. Its high-stakes nature stems from its direct inclusion in the high school admission score, typically accounting for 5–10% of the total score (Shi, 2018). Test items and scoring rubrics are standardized by local education authorities and often include the 50-meter sprint, 800m/1000m endurance run, standing long jump, pull-ups, sit-ups, and rope jumping. Students have little to no autonomy in selecting test items.

This standardized and compulsory framework creates tension with SDT's principle of autonomy. The PEEE significantly restricts student choice, preventing them from selecting activities aligned with their interests. Mandatory events such as the 800m run or pull-ups may replace preferred activities like basketball, badminton, or dance. This externally driven exam logic can lead students to view physical activity as task completion rather than enjoyable participation, reducing intrinsic motivation (Chen et al., 2014).

Furthermore, China's exam-oriented educational ecosystem amplifies these issues. Within this high-control environment, educational activities revolve around exams, and students' behavior is shaped by external incentives such as scores and promotion opportunities. When PE becomes an exam subject, students and parents often view it as part of competitive academic advancement rather than a means of health promotion or holistic development. Some students express negative or resistant attitudes toward the PEEE, feeling compelled to train daily, which increases academic and physical burdens and diminishes interest in physical activity (Liu and Wang, 2021).

Although the PEEE has achieved some positive outcomes—such as improvements in endurance performance and physical fitness indicators (Liu, 2008; Shi, 2018; Zhu et al., 2012)—these gains often result from short-term intensive training rather than sustained motivation, and tend to fade after the exam.

In contrast, countries such as the UK and Australia emphasize student autonomy and interest-based choices in PE assessment. For example, some Australian schools allow students to select from activities such as dance, swimming, or team sports. This flexibility better meets SDT's psychological needs and promotes intrinsic motivation (Sun et al., 2017).

Given these contrasts, China's PEEE provides a representative case for examining how compulsory assessments influence student motivation. Within a highly controlling exam-oriented culture, the exam not only serves health-promotion goals but is also tied to expectations of educational fairness and quality (Nie et al., 2021). This dual role creates tension between promoting exercise participation and managing exam pressure, challenging SDT's applicability in East Asian educational contexts.

Most existing Chinese studies focus on the exam's impact on physical fitness, with limited attention to deep psychological mechanisms, particularly intrinsic motivation and long-term participation (Xu and Ma, 2022; Zhou et al., 2017). Thus, investigating how the exam influences autonomy, competence, and relatedness—and, in turn, motivation and behavior—is crucial for filling this theoretical and empirical gap.

To address this gap, this study adopts a qualitative approach to explore core questions: How do students perceive the exam's compulsory nature and exam-oriented PE curriculum? How does the exam influence students' autonomy, competence, and relatedness? Does exam-driven instruction undermine interest and lifelong participation? How do teachers understand and respond to the exam's effects on student motivation and instructional practice? By examining these perspectives, the study aims to clarify the pathways through which the PEEE shapes students' motivational structures, provide theoretical support for policy reform, and extend SDT's explanatory power in non-Western contexts.

## 3 Methodology

### 3.1 Research design

To systematically examine how China's PEEE influences junior secondary students' motivation for physical education and their willingness for future participation in physical activity, this study adopts a qualitative research design grounded in Self-Determination Theory (SDT). The analytical focus is on how the exam system—embedded within a highly exam-oriented cultural context—shapes students' motivational types, psychological need satisfaction, and behavioral choices. Multiple data sources were employed, combining semi-structured in-depth interviews, focus group discussions, and classroom observations to achieve data triangulation and enhance the credibility and explanatory power of the findings. By comparing participants' narratives with actual teaching behaviors and students' engagement during lessons, this study aims to uncover how institutional pressures influence motivational internalization, teacher–student interactions, and students' practices in physical activity.

### 3.2 Participants and sampling strategy

Participants comprised junior secondary school students (mainly Grade 8 and Grade 9) and their physical education teachers in regions where the PEEE has been implemented and physical education scores count toward the high school admission score. Students were aged 12–15 and had either participated in or were preparing for the exam, thus possessing direct exam-related experience. Teachers were required to have at least 1 year of instructional experience related to the PEEE and to be familiar with exam items, scoring criteria, and recent policy adjustments.

A purposeful maximum variation sampling strategy was employed to maximize informational richness and contextual diversity. Four key dimensions guided participant selection: (1) regional economic development level (developed/moderately developed/less developed), (2) city size (large city/small city), (3) gender (male/female), and (4) participant role (teacher/student). Accordingly, four junior secondary schools were chosen as case sites: a large-city school in an economically developed eastern region (The Second Affiliated High School of East China Normal University, Shanghai), a small-city school in a moderately developed eastern region (Fenghua Middle School, Ningbo, Zhejiang), a large-city school in a moderately developed northeastern region (Fenghua Middle School, Harbin, Heilongjiang), and a small-city school in a less-developed western region (Changjiang School, Yibin, Sichuan).

Each school used purposeful sampling based on grade, gender, and exam preparation status. 6 students from each school were invited to participate in interviews or focus groups (total *n* = 24). In addition, two PE teachers with exam-related teaching experience were interviewed at each school (total *n* = 8). The overall sample size was controlled at a moderate level to balance data richness and theoretical saturation in thematic analysis.

### 3.3 Data collection

Data collection took place between September 8 and September 29, 2025, over a three-week period. With written informed consent from school administrators, teachers, students, and their guardians, the research team conducted student interviews, teacher interviews, focus group discussions, and classroom observations. Data saturation was determined when subsequent interviews and focus groups yielded no new themes or conceptual insights regarding students' motivational shifts.

The first are semi-structured in-depth interviews. The interview protocols were developed around the three core psychological needs of SDT—autonomy, competence, and relatedness—while addressing the central research question: “How does the PEEE influence students' motivation and willingness for future participation in physical activity?” Student interviews focused on: overall exam experience and emotional responses; sources of pressure during exam preparation; motivational types and internalization processes (e.g., “Do you train because you want to or because you have to?”); willingness to continue exercising after the exam; and potential changes in preferred sports. Example questions included: “Do you feel the PEEE is a motivation or a burden?” “If this exam did not exist, would you still exercise the way you do now?”

Teacher interviews explored the changes in teaching content and methods after exam implementation, perceived instructional pressure and responsibility, observed differences among student groups in motivation and engagement, strategies used to support students' motivation and confidence; and reflections on the exam items and assessment system. Example questions included: “How has your teaching changed since the implementation of the PEEE?” “Have you observed changes in students' engagement due to the exam?” Interviews were conducted in quiet on-campus locations, each lasting 45–70 min, and were audio-recorded with participant consent.

The second are focus group discussions. To capture group-level experiences, peer dynamics, and consensus/divergence, 4 student focus groups (3–4 students each) were conducted across the 4 schools. Groups were formed based on grade level, gender, and exam preparation stage to balance within-group diversity and between-group comparability. Each discussion lasted 60–90 min and was facilitated by one researcher, while another documented non-verbal cues. Discussion topics included: whether the exam is perceived as “pressure” or “motivation” among peers; whether exam preparation reshaped attitudes toward physical education; perceptions of fairness and choice in exam items; evaluations of teacher support and classroom climate; and suggestions for improving the exam and PE curriculum. Focus groups were particularly useful for capturing how students co-constructed narratives in peer settings and negotiated differing viewpoints.

The third are structured classroom observations. With school and parental consent, the research team observed 5–6 PE lessons across the case schools, including exam-focused training sessions and more flexible activity lessons. A structured observation checklist was used to systematically document: Autonomy support (e.g., whether students were offered choices; whether teachers used encouraging rather than controlling language); student engagement and emotional expressions (e.g., participation level, enthusiasm, anxiety, avoidance behaviors); classroom social climate (cooperation vs. competition; support vs. exclusion); types and frequency of teacher feedback (encouraging vs. critical feedback; individualized guidance vs. uniform demands).

Observation notes were later compared with interview data to identify consistencies or tensions between expressed beliefs and actual practices, thereby enhancing interpretive depth.

### 3.4 Data analysis

The study followed Braun and Clarke's (2006) six-step thematic analysis framework, using NVivo 12 for coding and theme development. The analytical steps included: (1) transcribing all audio recordings verbatim and repeatedly reading the transcripts to gain familiarity with the data; (2) conducting open initial coding of student and teacher interviews and observation notes, marking information related to exam experiences, motivational types, autonomy suppression, competence-related experiences, relatedness, external pressures, and instructional adjustments, while preserving participants' original wording where possible; (3) grouping conceptually similar codes into clusters and developing preliminary themes that reflected shared patterns; (4) reviewing themes in relation to the SDT framework, examining their logical coherence and distinctiveness, removing weak or overlapping themes, and refining those with unclear boundaries; (5) defining and naming core themes, clarifying their conceptual scope, and selecting representative participant quotations and classroom scenes as evidence; (6) using NVivo 12 to manage coding nodes, analytic memos, and theme structures to ensure transparency and traceability.

To enhance reliability, two researchers independently coded the data using a blind coding approach. Consistency between coders was ensured by calculating inter-coder agreement; any divergent interpretations were resolved through iterative discussion and re-examination of the raw transcripts until a 90% consensus was reached. The combination of multiple data sources, SDT-guided analysis, and this collaborative coding process strengthened the robustness and credibility of the findings, ensuring that the results accurately reflect the complex motivational landscape of the PEEE context.

### 3.5 Ethics and research trustworthiness

Prior to data collection, ethical approval was obtained from the relevant institutional review board, and written informed consent was secured from participating schools, teachers, students, and their guardians. Participants were informed of the study purpose, data use, and their right to withdraw at any time. All interview and observation data were anonymized during transcription, with identifiable information removed or replaced by codes. Raw recordings and text files were stored on encrypted devices accessible only to the research team. Trustworthiness was enhanced through data triangulation, inter-coder agreement, and thick description to support the credibility and transferability of the findings.

## 4 Findings

Drawing on in-depth interviews with 8 PE teachers and 24 students from 4 schools, supplemented by classroom observations, this study systematically analyzes how the PEEE shapes students' motivation for physical education from the perspective of the three basic psychological needs in Self-Determination Theory. Five core themes emerged: constrained autonomy, the dual construction of competence, the functional distortion of relatedness, the predominance of extrinsic motivation, and teachers' dual pressures. Taken together, students' and teachers' narratives show that the PEEE is reshaping the practical logic of school PE, the structure of teacher–student relationships, and students' motivational experiences, revealing marked and complex cultural and institutional tensions.

### 4.1 Constrained autonomy: “Physical Action” defined by the exam

Under the PEEE regime, students commonly experience a sharp reduction in autonomy in PE classes. Almost all interviewees mentioned that PE has increasingly become an “exam-preparation field” rather than a space for free choice, interest exploration, and the enjoyment of movement. In students' narratives, expressions such as “no choice,” “have to practice,” “can only practice exam events,” and “if you don't practice you'll lose marks” appeared frequently, indicating that participation in physical activity is strongly shaped by institutionalized goals.

At Shanghai Huashida No. 2 (ECNU Second Affiliated), a girl (Student S1) said: “Actually, many students in our class like activities such as dance, badminton, or yoga, but these are almost never arranged in PE lessons. We basically just practice running, rope skipping, sit-ups—everything revolves around the exam. Every time I go to PE, I don't feel like I'm in a class; I feel like I'm in exam prep.”

She further explained that although teachers occasionally wanted to organize fun activities, as soon as they realized “there are only 2 months left before the exam,” both teachers and students would automatically pull the curriculum back to lap-running and repetitive test-focused drills, as if all other forms of exercise had lost their legitimacy. A boy at Harbin Fenghua Middle School (S14) expressed similar frustration: “I used to love playing basketball with my classmates in PE. That happiness is completely gone now. The PE teacher says, ‘We'll play after the exam,' but after the exam it's summer vacation, and later they won't let us play either. I feel like PE has lost its soul—it's just compulsory practice for the exam items.”

Teacher interviews further revealed the structural reasons behind the loss of autonomy. A teacher from Ningbo (T3) admitted: “Of course PE teachers want the curriculum to be more diverse, but the school has hard targets for PEEE scores, and parents are watching. If the scores are not good, they come to question the teacher, so we basically don't dare to teach anything else.” A teacher from Changjiang School in Yibin (T8) went even further: “If I arrange free activities for students, the school will think I'm ‘wasting time.' I want them to experience more kinds of sport, but with the pressure of high school admissions there, I can only guarantee scores first.” In this highly controlling institutional environment, even teachers who recognize the importance of autonomy often find themselves unable to implement it.

Notably, many students stated that the PEEE has not only changed their choices within class but also reshaped their overall understanding of physical activity. A boy from Ningbo (S10) said: “Now we exercise for the sake of the exam, not for interest. For example, I like swimming, but because swimming doesn't count toward the score, I don't dare spend too much time on it. You feel that what you like doesn't matter; what matters is what the exam wants.” This shift in motivational structure indicates that students' physical actions have moved from being “interest-based” to “task-based.” The meaning of exercise has shifted from “personal experience” to “exam requirement,” and students' behavioral space has become deeply embedded in a highly externally controlling educational context.

From an SDT perspective, prolonged frustration of autonomy needs leads to resistance toward the activity, emotional depletion, and the externalization of motivation. The interview data clearly show that standardized exam items and admission-oriented resource allocation leave students almost no room for choice in PE. As a result, physical activity is reframed as an “externally prescribed action,” which triggers a mechanism of external regulation where students comply only to avoid negative consequences. This systemic constraint prevents the integration of exercise into the student's core identity, thereby weakening their sense of agency and stripping the activity of any personal meaningfulness beyond its utility as a score-getting tool.

### 4.2 The dual construction of competence: coexistence of achievement and withdrawal

The PEEE produces a striking polarization in students' sense of competence. Some students experience clear improvement through sustained practice and thus feel achievement and self-confidence arising from enhanced physical capabilities. Others, however, due to innate differences, the difficulty of events, training-related suffering, and repeated failure, gradually develop a strong sense of helplessness and even fear of PE, with some explicitly describing it as a “class of frustration.”

Many “successful” students described positive experiences brought about by the PEEE. A boy from Ningbo (S8) recalled: “I couldn't finish the 1,000 meters before, but after practicing for the past 2 years I've really become much faster—from more than 5 min down to 4 min 10. I feel like I've really become stronger. You can literally see your hard work pay off.” He saw this as “a sense of achievement brought by PE.” A girl from Yibin (S21) similarly said: “The teacher corrected my movements and taught me the rhythm. I went from 120 skips to 180 and then over 200. That feeling of improving day by day is really great.”

These narratives show that when schools provide reasonable instructional methods, appropriate feedback, and attainable goals, students' competence can be strengthened, and motivation can gradually shift from external pressure toward internal endorsement.

By contrast, the narratives of “unsuccessful” students were more numerous and often more intense. At Harbin Fenghua, a girl (S15) described her suffering with running: “I really can't run fast, but in our school the 800 meters is super important. Everyone is watching. Every time I run, the teacher calls me out and criticizes me. I feel like no matter how I practice, it's useless. Now I get a headache whenever I hear that we're going to run.” A boy (S16) added: “For things like pull-ups, even if you train for a year you might not manage one more rep. I get zero every time on the test, so I just gave up. You feel like you're not cut out for sports.”

These students frequently mentioned an “unbridgeable gap” between standardized exam requirements and their physical conditions. Effort and outcome could not be reliably linked, and a cumulative sense of failure grew over time.

Teachers were also acutely aware of this “competence differentiation.” A teacher from Shanghai (T2) observed: “Talent gaps in PE are even more obvious than in academic subjects, especially in endurance and strength events. Some children don't improve even after running ten times. They start feeling PE is not their strength and even grow to dislike PE lessons.” A teacher from Ningbo (T3) added: “If the class requirements are strict, weaker students suffer more. Making them train every day actually eats away at their interest in PE.” Teachers generally believed that the PEEE had eroded the educational value of PE as a space for health and enjoyable experience, trapping many students in a cycle of failure.

Some teachers, however, tried to repair students' competence through individualized support. A girl from Ningbo (S9) shared: “I completely crashed in my 800-meter run. The teacher walked with me for a long time and told me, ‘It's okay, we'll take it slowly.' Later he helped me practice breathing and pacing every day. During that period I really felt he was trying to help me, and that feeling of being cared about gave me the courage to keep trying.” Such support not only improved her performance but also rebuilt her sense of competence at a psychological level.

Overall, the PEEE constructs competence through a mechanism of performance-contingent validation, which manifests in a highly differentiated way. For some students, it forms a positive chain of “effort → progress → achievement”; for others, it creates a negative chain of “difficulty → failure → withdrawal.” These chains coexist because the exam-oriented structure forces students to derive their sense of competence from standardized benchmarks rather than personal mastery. This underlying mechanism suggests that competence in this context is often “fragile,” as it is tied to high-stakes outcomes rather than an internalized belief in one's physical potential. This underscores the crucial impact of event characteristics and assessment design on shaping whether students perceive their efficacy as a sustainable resource for growth or a source of evaluative pain.

### 4.3 The functional distortion of relatedness: less cooperation, more competition

Physical activity is inherently social, yet under the PEEE, students report a marked decline in the quality of social relationships in PE. They no longer experience PE lessons as spaces for “peer cooperation, team participation, and mutual companionship,” but rather as arenas of “individual practice, personal competition, and score stratification.” Peer relations shift from mutual help to comparison; teacher–student relations shift from companionship to supervision; and the class climate moves from shared experience to individualized score-seeking.

Most students felt that the social atmosphere in PE had undergone a structural change. A boy in Shanghai (S5) described it vividly: “In the past, PE was the whole class doing activities together. Now everyone is practicing their own event—you do your skipping, I do my sit-ups—there's basically no cooperation.” A girl from Harbin (S18) added: “Everyone is fighting for scores, so they don't really want to help others. It's not that they don't want to help, it's that they're afraid of wasting time.” This shows that within a high-stakes evaluation system, “helping others” is interpreted as a non-profitable behavior, and cooperative actions struggle to find a place within the logic of score maximization.

Teachers also observed changes in peer relationships. A teacher from Ningbo (T3) noted: “In the past, children would cheer each other on. Now many just focus on themselves. They compare scores with each other and sometimes even argue over who skipped more.” A teacher from Yibin (T8) mentioned: “Some students who are weaker get isolated. They themselves feel like they're holding others back, so they're even less willing to join group activities.”

Teacher–student relationships have also shifted. Many students described teachers as “stricter” and “more like referees.” A boy from Harbin (S17) commented: “The teacher now mainly uses their energy to supervise us—timing, counting, correcting movements. It feels like the teacher isn't ‘someone exercising with us' but ‘someone checking whether we meet the standard.”' A girl from Yibin (S22) stated even more directly: “The teacher is really busy now. They have to record scores, make forms, and pick key students for extra coaching. You feel like they don't really have time to pay attention to you.”

Classroom observations confirmed this “exam-ification of interaction patterns”: teachers spent most of their time patrolling, correcting, and recording, leaving little space for group activities or more personal interaction. Still, a few teachers tried to preserve emotional connection despite the pressure. A girl from Ningbo (S11) shared: “Our teacher is relatively gentle. When we're exhausted from running, he sometimes runs alongside us, saying, ‘I'll run with you.' That feels really good.” This kind of “running together” support can significantly enhance students' sense of belonging and trust, providing important emotional backing.

Overall, the PEEE transforms the relational logic of PE from “cooperation and participation” to “competition and comparison,” thereby weakening the satisfaction of students' need for relatedness. PE is no longer primarily a way for students to build emotional connections with peers and teachers; it becomes a competitive arena for scores. This shift triggers a mechanism of “Social Alienation,” where peers are perceived as benchmarks for comparison rather than sources of support. From an SDT perspective, this erosion of relatedness blocks the emotional pathway to internalization, as the lack of a secure and supportive social climate makes it difficult for students to transition from external pressure to a personal valuing of physical activity. This not only damages the social function of PE but also creates a motivational vacuum that suppresses long-term participation.

### 4.4 Predominance of extrinsic motivation: PE as a “score-getting tool”

Within the PEEE context, students' motivation to participate in physical activity is heavily “externally controlled.” Their responses revolve around “scores, admission, tasks, and coercion,” rather than “interest, enjoyment, health, or meaning.” Extrinsic motivation has clearly become dominant.

Students almost unanimously stated that the purpose of “doing PE” is not health or interest but scores. A girl from Shanghai (S3) said bluntly: “I don't like running, but I have to run because the PE score is so high—it directly affects the total score.” A boy from Ningbo (S12) remarked: “For me, a few more points are just a few more points. PE is simply one subject for high school entrance. There's nothing more to say about it.” A boy from Yibin added: “PE is the easiest subject to boost your score. If you don't train, you're just losing out.”

Many students explicitly said they would stop training immediately after the exam. A boy from Yibin (S24) shared: “After I finished the PE exam, I didn't want to run at all for a week—like I'd just woken up from a nightmare.” Another boy (S13) stated: “Running, for me, is only related to the exam. Without the exam, I'd never run.” These accounts reveal a clear “backfire effect”: extrinsic motivation not only fails to transform into intrinsic motivation but even leads to aversion and rejection of the activity itself.

The role of parents and schools in pushing extrinsic motivation was frequently mentioned. A teacher from Shanghai (T2) explained: “Parents now enroll their children in private PE coaching just to get points. Whether the child likes it or not, they don't really care; they only care about the score.” A teacher from Ningbo (T3) added: “The school treats PE scores as a key performance indicator. If a class has low PE scores, it affects performance evaluations. All this pressure ultimately gets passed down to the students.” These comments show how the entire school system instrumentalizes PE for score production, further reinforcing students' extrinsic motivation.

In sum, the PEEE ties physical activity tightly to admission pressure, redefining exercise as a means to “gain points.” Its inherent health, enjoyment, and social values are marginalized, creating a “crowding-out effect” where the dominant utilitarian purpose stifles the development of personal interest. This shift disrupts the internalization process, making it increasingly difficult for students to move along the motivational continuum from external regulation toward integrated or intrinsic motivation. As exercise becomes an instrument of the exam system rather than a self-determined choice, the psychological foundation for sustained participation beyond the testing period is significantly undermined.

### 4.5 Teachers' dual pressures: pulled between educational ideals and admission reality

In implementing the PEEE, teachers experience persistent dual pressures. On one hand, they hope PE can truly embody the educational principle of “health first,” cultivating students' interest and lifelong exercise habits. On the other hand, institutional structures, parental expectations, and score-based accountability compel them to act as “trainers” and “score managers.” Their narratives are filled with feelings of powerlessness, inner conflict, and being forced to change.

A teacher in Shanghai (T1) expressed deep frustration: “From the perspective of PE education, we should let children experience more types of sport and cultivate their interest. But parents and schools only care about scores. If you don't teach exam events, parents will question you.” A teacher in Harbin (T5) similarly noted: “I want to let the kids play ball, but the principal says, ‘This is a critical period, you must secure the scores.' So I can only comply.”

Teachers' professional judgments are often marginalized under institutional pressure. In daily teaching, teachers also feel exhausted and reactive. A teacher from Yibin (T7) said: “PE teachers now aren't really ‘teaching PE'; they're making ‘training plans'—arranging events, dividing groups, organizing drills, recording scores every day. It's like we're coaching a professional team.” They reported that lessons have become monotonous, training-oriented, and mechanical, leading to a decline in lesson quality.

Teachers also face pressure from parents. A teacher in Ningbo (T4) commented: “Some parents ask me, ‘Other kids can skip 200 times—why can't my child?' They push all the responsibility onto the teacher.” A teacher in Harbin (T6) added: “When students get injured during training, parents complain, but they still want their child to get high scores. It's really hard for teachers.” These pressures are a major source of teacher burnout.

Nevertheless, some teachers still try to preserve the professionalism and humanistic value of PE within the restricted space they have. They incorporate encouragement and emotional support into training, striving to prevent students from losing interest under high pressure. A girl from Shanghai (S2) recalled emotionally: “The teacher saw me crying while I was running and deliberately walked with me around the track, telling me I had already tried very hard and that it was okay if I didn't do well. That day I felt that PE teachers can be really warm.”

This kind of emotional labor is particularly precious amid systemic pressure. Many teachers believe that without exam pressure, they would use more open, diverse, and participatory teaching approaches, allowing students to experience a wider range of sports rather than being constrained to a few items such as standing long jump, sit-ups, and endurance running. They hope that future PE assessment reforms will reduce uniform exam requirements and grant more educational space to PE lessons.

Overall, teachers are caught in multiple tensions within the exam system: they must meet policy targets yet want to realize educational ideals; they must secure student scores yet wish to protect students' interest; they must respond to school performance metrics yet bear parents' expectations. They are not only policy implementers but also emotional laborers and guardians of educational values. Their working conditions have a direct impact on the formation and erosion of students' motivation for physical activity.

## 5 Conclusion and discussions

Using Self-Determination Theory as the analytical framework, this study systematically analyzed interview, focus group, and classroom observation data from teachers and students in schools across different regions and contexts, with the aim of exploring how a high-stakes mechanism such as the PEEE shapes students' motivation for physical activity within China's exam-oriented education system. The findings show that, to some extent, the PEEE has increased the importance attached to PE by schools and families and has enabled some students to improve their physical fitness and gain short-term confidence through structured training. However, in practice, its deeper impact lies in the restriction of students' basic psychological needs: autonomy is compressed by highly structured exam content; competence becomes sharply differentiated due to ability gaps; relational support is weakened in a competitive environment. Crucially, the high-stakes nature of the exam disrupts the motivational internalization process; instead of moving from external regulation toward identified or integrated regulation, students' motivation remains heavily externalized and “score-driven,” making it difficult to sustain exercise once the external pressure is removed. The study contributes to SDT by illustrating how a “controlling” institutional culture can lead to a “functional distortion” of psychological needs, where even observed gains in competence fail to foster an autonomous active identity. The conflicting situation faced by teachers—between their educational ideals and admission pressures—further illustrates the complexity and tension inherent in the system's operation.

Overall, while China's PEEE contributes to the policy goal of improving physical fitness, it also entails significant educational costs. The theoretical contribution of this study lies in its extension of SDT into a high-stakes, non-Western institutional context, revealing how exam-oriented culture creates a “functional distortion” of psychological needs. It clarifies that in such systems, competence gains do not automatically lead to motivation internalization unless autonomy is concurrently protected. Practically, this research moves beyond general criticism to provide specific implications for reform. For teaching practice, it suggests shifting from “test-centric” coaching to “autonomy-supportive” pedagogy, such as providing meaningful rationales for drills and incorporating student-led goal setting to foster a sense of ownership. At the policy level, we recommend a transition from a rigid, “one-size-fits-all” testing model to a “portfolio-based” or “process-oriented” assessment system that rewards individual progress and diverse sporting interests. This analysis not only illuminates the psychological mechanisms of PE motivation in the Chinese context but also provides a roadmap for constructing an assessment system that balances fairness with humanistic values. By providing students with more differentiated assessment approaches and supportive learning environments, future reforms can better advance the ultimate goals of PE: the transformation of “mandatory fitness” into lifelong participation and holistic development.

Of course, this study has several limitations. First, participants were mainly Grade 8 and Grade 9 students, resulting in a relatively narrow age range. It was therefore not possible to fully capture differences in how students at different developmental stages understand the PE assessment system. Second, while the qualitative design allowed for an in-depth exploration of the emotional and experiential worlds of students and teachers, the sample size and regional coverage remain limited, making it difficult to generalize findings to the national level. Third, the study focused primarily on the subjective experiences of students and teachers, paying less attention to the policy-making logic behind the PEEE, school-level organizational mechanisms, and forms of parental involvement. As a result, the broader ecological structure within which motivation is formed is only partially presented.

In light of these limitations, future research could be extended in at least three directions. First, large-scale quantitative studies could be conducted to measure differences in autonomy, competence, and relatedness among students from different regions and school types, thereby enhancing the generalizability and applicability of the findings. Second, longitudinal studies could trace students' trajectories of physical activity from exam preparation through senior high school to more accurately assess the long-term effects of the PEEE on exercise behavior. Third, future work could incorporate multiple actors—such as school administrators, policymakers, and parents—to build a more comprehensive ecological analytic framework that reveals the value orientations, governance logics, and sociocultural factors underlying the mechanism of the PEEE. In addition, comparative research on non-exam-oriented PE assessment systems abroad could enrich the theoretical perspectives available for reforming China's PE evaluation system.

## Data Availability

The original contributions presented in the study are included in the article/Supplementary material, further inquiries can be directed to the corresponding author.

## References

[B1] Bai Y. Nie R. Li X. (2022). Historical changes, evolutionary characteristics and future direction of physical education reform in China's high school entrance examination. China Examinations, (3), 15–23. Available online at: https://www.xinhuanet.com/politics/2021-03/10/c_1127194872.htm (Accessed October 03, 2021).

[B2] Braun V. Clarke V. (2006). Using thematic analysis in psychology. Qual. Res. Psychol. 3, 77–101. doi: 10.1191/1478088706qp063oa

[B3] Chen X. Wang J. Yan F. (2014). The relationship between adolescents' aesthetic tendencies in sport and their judgments of sport values: evidence from Zhejiang province. J. Sports Sci. 34, 29–38. doi: 10.16469/j.css.2014.03.004

[B4] Cox A. E. Smith A. L. Williams L. (2008). Change in physical education motivation and physical activity behavior during middle school. J. Adolesc. Health 43, 506–513. doi: 10.1016/j.jadohealth.2008.04.020 18848680

[B5] Deci E. L. Ryan R. M. (1985). Intrinsic Motivation and Self-Determination in Human Behavior. New York, NY: Plenum Press. doi: 10.1007/978-1-4899-2271-7

[B6] Deci E. L. Ryan R. M. (2000). The “what” and “why” of goal pursuits: human needs and the self-determination of behavior. Psychol. Inq. 11, 227–268. doi: 10.1207/S15327965PLI1104_0133716585

[B7] Deci E. L. Ryan R. M. (2008). Self-determination theory: a macrotheory of human motivation, development, and health. Can. Psychol. 49, 182–185. doi: 10.1037/a0012801

[B8] Hagger M. Chatzisarantis N. (2008). Self-determination theory and the psychology of exercise. Int. Rev. Sport Exerc. Psychol. 16, 26–28. doi: 10.1080/17509840701827437

[B9] Hagger M. S. Chatzisarantis N. L. D. Barkoukis V. Wang C. K. J. Baranowski J. (2005). Perceived autonomy support in physical education and leisure-time physical activity: a cross-cultural evaluation of the trans-contextual model. J. Educ. Psychol. 97, 376–390. doi: 10.1037/0022-0663.97.3.376

[B10] Liu H. (2008). An analysis of the implementation status of the physical education examination system for junior high school graduation and admission in China. J. Phys. Educ. 9, 8–14. doi: 10.16237/j.cnki.cn44-1404/g8.2008.09.001

[B11] Liu Y. Wang F. (2021). How far do we still have to go? – Deputies and members discuss the reform of PE in the high school and secondary school entrance examinations. Xinhua News. Available online at: https://www.xinhuanet.com/2021-03/10/c_1127194872.htm (Accessed June 10, 2025).

[B12] Ministry of Education of the People's Republic of China, (2020). Opinions on Comprehensively Strengthening and Improving School Physical Education in the New Era. Available online at: http://www.moe.gov.cn/jyb_xxgk/moe_1777/moe_1778/202010/t20201015_494794.html (Accessed October 5, 2020).

[B13] Nie Z. Liu J. Gao F. (2021). Reform of the PE entrance examination: origins, values, and pathways. J. Beijing Sport Univ. 44, 76–85. doi: 10.19582/j.cnki.11-3785/g8.2021.09.008

[B14] Ntoumanis N. (2001). A self-determination approach to the understanding of motivation in physical education. Br. J. Educ. Psychol. 71, 225–242. doi: 10.1348/000709901158497 11449934

[B15] Reeve J. (2009). Why teachers adopt a controlling motivating style toward students and how to become more autonomy supportive. Educ. Psychol. 44, 159–175. doi: 10.1080/00461520903028990

[B16] Ryan R. M. Deci E. L. (2000). Self-determination theory and the facilitation of intrinsic motivation, social development, and wellbeing. Am. Psychol. 55, 68–78. doi: 10.1037/0003-066X.55.1.68 11392867

[B17] Ryan R. M. Deci E. L. (2017). Self-Determination Theory: Basic Psychological Needs In Motivation, Development, And Wellness. New York, NY: *The Guilford Press*. doi: 10.1521/978.14625/28806

[B18] Shi W. (2018). A panoramic study of the 2017 national physical education entrance examination. J. Beijing Sport Univ. 41, 90–96. doi: 10.19582/j.cnki.11-3785/g8.2018.08.014

[B19] Sparks C. Dimmock J. D. Donsdale C. Jackson B. (2016). Modeling indicators and outcomes of students' perceived teacher relatedness support in high school physical education. Psychol. Sport Exerc. 26, 71–82. doi: 10.1016/j.psychsport.2016.06.004

[B20] Spray C. M. Wang C. K. J. (2001). Goal orientations, self-determination and pupils' discipline in physical education. J. Sports Sci. 19, 903–913. doi: 10.1080/026404101317108417 11820685

[B21] Sun H. Li W. Shen B. (2017). Learning in physical education: sself-determination theory perspective. J. Teach. Phys. Educ. 36, 277–291 doi: 10.1123/jtpe.2017-006734173186

[B22] Taylor I. M. Ntoumanis N. Standage M. Spray C. M. (2010). Motivational predictors of physical education students' effort, exercise intentions, and leisure-time physical activity: a multilevel linear growth analysis. J. Sport Exerc. Psychol. 32, 99–120. doi: 10.1123/jsep.32.1.99 20167954

[B23] Vansteenkiste M. Lens W. Deci E. L. (2006). Intrinsic versus extrinsic goal contents in self-determination theory: another look at the quality of academic motivation. Educ. Psychol. 41, 19–31. doi: 10.1207/s15326985ep4101_433829831

[B24] Vansteenkiste M. Van Petegem S. Wuyts V. (2010). Pathways to student motivation: a meta-analysis of antecedents of autonomous and controlled motivations. Rev. Educ. Res. 80, 304−337. doi: 10.3102/0034654310377070 PMC8935530 35330866

[B25] Xu H. Ma L. (2022). Research on the development trends of PEEE reform under the “Double Reduction” policy context. J. Tianjin Univ. Sport 37, 38–43. doi: 10.13297/j.cnki.issn1005-0000.2022.01.006

[B26] Zhou H. Gu Y. Liu X. (2017). The “hot effects” and “cold reflections” on school physical education under the background of PE entrance exam reform. J. Beijing Sport Univ. 40, 68–75. doi: 10.19582/j.cnki.11-3785/g8.2017.07.012

[B27] Zhu L. Xu Y. Liu L. (2012). The influence of the PE entrance exam system on school physical education and countermeasure research. J. Chengdu Sport Univ. 38, 86–89. doi: 10.15942/j.jcsu.2012.04.021

