# Supplementary material for: The impact of mandatory physical fitness testing on students' intrinsic motivation and sustained participation in physical activity: a self-determination theory perspective
Source: Front Psychol. 2026 Jun 10;17:1821930. doi: 10.3389/fpsyg.2026.1821930 (PMC13291134; doi:10.3389/fpsyg.2026.1821930)
Supplement: Supplementary file 1 [file Data_Sheet_1.docx]

**The Impact of Mandatory Physical Fitness Testing on Students’ Intrinsic Motivation and Sustained Participation in Physical Activity:**

**A Self-Determination Theory Perspective**

Ying Chen

Associate Professor

School of Physical Education and Health

East China Normal University

Jiahang Jiang

Student

School of Physical Education and Health

East China Normal University

Wenwu Liu

Professor

School of Physical Education and Health

East China Normal University

**Abstract**

**Introduction:** With physical education included in China’s high school entrance examination, the high-stakes Physical Education Entrance Examination (PEEE) has altered school physical education arrangement. Previous studies mainly focus on physical fitness outcomes but lack in-depth exploration of motivational mechanisms from Self-Determination Theory (SDT). This research aims to examine how PEEE influences students’ sport motivation and sustained physical activity participation by affecting their three basic psychological needs of autonomy, competence and relatedness.

**Methods:** A qualitative approach was employed. Twenty-four junior high students and eight PE teachers from four geographically diverse middle schools were selected via purposive sampling. Data were collected through individual semi-structured interviews, focus group talks and classroom observations, and thematic analysis based on Braun and Clarke’s framework was conducted with NVivo 12 software.

**Results:** The PEEE increases institutional and parental attention to PE and improves short-term physical fitness and self-confidence for part of students. However, rigid exam-oriented courses limit students’ autonomous choices; individual physical gaps result in polarized feelings of competence; score competition erodes supportive interpersonal relatedness. Most students form extrinsic exercise motivation instead of internalized interest, and teachers face persistent conflicts between educational ideals and exam assessment pressure.

**Discussion:** Although the PEEE partially achieves fitness promotion goals, it distorts three core psychological needs proposed by SDT and blocks the internalization of sport motivation. To foster lifelong exercise behavior, future PE reform should replace unified high-stakes testing with diversified process evaluation and adopt autonomy-supportive teaching strategies.

**Keywords:** Physical Education Entrance Examination; Self-Determination Theory; Autonomy; Competence; Relatedness

**Appendix**

## A. List of Student Interview Participants (S1–S24)

| ***ID*** | ***Gender*** | ***Grade*** | ***School*** | ***Type of Participation*** |
| --- | --- | --- | --- | --- |
| S1 | Female | 9 | SAHS | In-Depth Interview |
| S2 | Female | 9 | SAHS | Focus Group + In-Depth Interview |
| S3 | Female | 9 | SAHS | Focus Group + In-Depth Interview |
| S4 | Male | 9 | SAHS | In-Depth Interview |
| S5 | Male | 8 | SAHS | Focus Group |
| S6 | Male | 9 | SAHS | Focus Group |
| S7 | Male | 9 | NFMS | In-Depth Interview |
| S8 | Male | 9 | NFMS | In-Depth Interview |
| S9 | Female | 8 | NFMS | Focus Group + In-Depth Interview |
| S10 | Male | 9 | NFMS | In-Depth Interview |
| S11 | Female | 9 | NFMS | Focus Group |
| S12 | Male | 8 | NFMS | Focus Group |
| S13 | Male | 8 | HFMS | In-Depth Interview |
| S14 | Male | 9 | HFMS | In-Depth Interview |
| S15 | Female | 9 | HFMS | Focus Group + In-Depth Interview |
| S16 | Male | 9 | HFMS | In-Depth Interview |
| S17 | Male | 8 | HFMS | Focus Group |
| S18 | Female | 9 | HFMS | Focus Group |
| S19 | Female | 9 | YCJS | In-Depth Interview |
| S20 | Male | 9 | YCJS | In-Depth Interview |
| S21 | Female | 8 | YCJS | Focus Group + In-Depth Interview |
| S22 | Female | 9 | YCJS | Focus Group + In-Depth Interview |
| S23 | Female | 9 | YCJS | Focus Group |
| S24 | Male | 8 | YCJS | Focus Group |

**Notes**: (1) Grade 8 is the second year of China’s junior secondary school; (2) Grade 9 is the final year of China’s junior secondary school; (3) SAHS refers to The Second Affiliated High School of East China Normal University, Shanghai; (4) NFMS refers to Fenghua Middle School, Ningbo, Zhejiang; (5) HFMS refers to Fenghua Middle School, Harbin, Heilongjiang; (6) YCJS refers to Changjiang School, Yibin, Sichuan

##

## B. List of Teach Interview Participatants（T1–T8）

| ***ID*** | ***Gender*** | ***Years of Teaching Experience*** | ***School*** | ***Type of Participation*** |
| --- | --- | --- | --- | --- |
| T1 | Male | 10 | SAHS | In-Depth Interview |
| T2 | Female | 8 | SAHS | In-Depth Interview |
| T3 | Female | 12 | NFMS | In-Depth Interview |
| T4 | Male | 6 | NFMS | In-Depth Interview |
| T5 | Male | 3 | HFMS | In-Depth Interview |
| T6 | Female | 9 | HFMS | In-Depth Interview |
| T7 | Male | 15 | YCJS | In-Depth Interview |
| T8 | Male | 5 | YCJS | In-Depth Interview |

**Notes:** (1) SAHS refers to The Second Affiliated High School of East China Normal University, Shanghai；(2) NFMS refers to Fenghua Middle School, Ningbo, Zhejiang; (3) HFMS refers to Fenghua Middle School, Harbin, Heilongjiang; (4) YCJS refers to Changjiang School, Yibin, Sichuan.

**C. Interview Questions for Students**

### I. Basic Information

- Grade, gender
- Whether they have taken or are preparing for the Physical Education (PE) High School Entrance Examination
- Preferred or regularly practiced physical activities

Theme 1: Experiences and Emotional Responses to the PE Entrance Examination

1. What do you think of the PE entrance examination? What words would you use to describe it?
2. Do you feel stressed during the preparation for the exam? Where does the pressure mainly come from?
3. How is the training for the exam different from your regular PE classes? Do you like this type of training?

Theme 2: Motivation and Willingness to Participate (Focusing on Internal vs. External Motivation)

1. When you practice the exam items, is it more because you *want* to, or because you *have* to?
2. Do you think the PE exam has increased your interest in physical exercise? Why or why not?
3. If there were no PE entrance examination, would you still continue exercising? Would the type of activities you choose change?

Theme 3: Sense of Autonomy and Choice

1. Did you choose the exam items yourself? Are you interested in these items?
2. If you could choose your own PE exam items, what would you pick? Why?
3. In PE class, do you feel you have room to make choices? Do you ever feel forced?

Theme 4: Sense of Competence and Teacher Support

1. Do you think you can successfully complete the exam items? Are you confident?
2. How does your teacher help you during training? Do you feel supported?
3. Has there been a time when you felt a strong sense of achievement? Or a time when you felt frustrated or embarrassed?

Theme 5: Sense of Belonging and Social Relationships

1. During PE training, is there more competition or mutual support among classmates?
2. In PE class, do you feel that the teacher is “on your side”?
3. What changes would make you more willing to participate in PE classes?

**D. Interview Questions for Teachers**

### Basic Information

- Years of teaching experience; whether they teach PE exam items
- Whether they have participated in PE exam reforms or teaching adjustments

Theme 1: Teaching Practices and Adaptation to Policy

1. Has your teaching content or style changed since the implementation of the PE entrance exam? In what ways?
2. Do you feel more teaching pressure than before? Where does it mainly come from?
3. During training and teaching, do you have room to address students’ individual interests and differences?

Theme 2: Student Motivation and Classroom Performance

1. Do you think the PE exam motivates or discourages students’ participation? Why?
2. Which types of students are most likely to train hard because of the exam? Which types tend to resist?
3. Do students practice on their own? Are they consistently interested in physical activities?

Theme 3: Teacher Role and Supporting Student Motivation

1. Do you consciously try to enhance students’ confidence and positive feelings? How?
2. In your teaching, do you emphasize discipline or interest more? How do you view this balance?
3. Have you observed any changes in students’ motivation related to the PE exam? Please give examples.

Theme 4: Evaluation System and Suggestions for Improvement

1. Do you think the current exam items and standards are scientific and reasonable? Why or why not?
2. If you had the opportunity to adjust the system, how would you improve the exam format or teaching arrangements?
3. What kind of PE curriculum or assessment system would better help students maintain long-term interest in exercise?

**E. Focus Group Questions for Students**

### Module 1: Overall Perceptions and Feelings

- Do you think the PE exam is “pressure” or “motivation”? What are the different opinions in the group?
- Does anyone particularly like one of the exam items? Why?

Module 2: Training Process and Motivation

1. When you attend PE class, is it mainly for exercise, for exam scores, or for other reasons?
2. Has anyone continued exercising after taking the exam? What activities did you choose and why?

Module 3: Fairness and Sense of Choice in the Exam System

1. Do you think the PE exam is “fair”? Why or why not?
2. If you could choose your own exam items, what would you add or remove?

Module 4: Teacher Support and Classroom Climate

1. During training, does the teacher feel more like a “coach” or a “referee”? Which do you prefer?
2. Have you ever felt encouraged or noticed in PE class? Can you share an example?

Module 5: Suggestions and Future Expectations

1. If you could write a letter to the education bureau suggesting improvements to the PE exam, what would you say?
2. What do you think an ideal PE class should be like?
